# Age-Modulated Associations between *KIBRA*, Brain Volume, and Verbal Memory among Healthy Older Adults

**DOI:** 10.3389/fnagi.2017.00431

**Published:** 2018-01-10

**Authors:** Ariana Stickel, Kevin Kawa, Katrin Walther, Elizabeth Glisky, Ryan Richholt, Matt Huentelman, Lee Ryan

**Affiliations:** ^1^Cognition and Neuroimaging Laboratory, Department of Psychology, University of Arizona, Tucson, AZ, United States; ^2^Epilepsy Center Erlangen, Department of Neurology, University Hospital Erlangen, Erlangen, Germany; ^3^Aging and Cognition Laboratory, Department of Psychology, University of Arizona, Tucson, AZ, United States; ^4^Neurogenomics Division, The Translational Genomics Research Institute, Phoenix, AZ, United States

**Keywords:** *KIBRA*, brain volumes, age-interactions, cognition, resource modulation

## Abstract

The resource modulation hypothesis suggests that the influence of genes on cognitive functioning increases with age. The *KIBRA* single nucleotide polymorphism rs17070145, associated with episodic memory and working memory, has been suggested to follow such a pattern, but few studies have tested this assertion directly. The present study investigated the relationship between *KIBRA* alleles (T carriers vs. CC homozygotes), cognitive performance, and brain volumes in three groups of cognitively healthy adults—middle aged (ages 52–64, *n* = 38), young old (ages 65–72, *n* = 45), and older old (ages 73–92, *n* = 62)—who were carefully matched on potentially confounding variables including apolipoprotein ε4 status and hypertension. Consistent with our prediction, T carriers maintained verbal memory performance with increasing age while CC homozygotes declined. Voxel-based morphometric analysis of magnetic resonance images showed an advantage for T carriers in frontal white matter volume that increased with age. Focusing on the older old group, this advantage for T carriers was also evident in left lingual gyrus gray matter and several additional frontal white matter regions. Contrary to expectations, neither *KIBRA* nor the interaction between *KIBRA* and age predicted hippocampal volumes. None of the brain regions investigated showed a CC homozygote advantage. Taken together, these data suggest that *KIBRA* results in decreased verbal memory performance and lower brain volumes in CC homozygotes compared to T carriers, particularly among the oldest old, consistent with the resource modulation hypothesis.

## Introduction

Since the initial *KIBRA* study demonstrating a benefit of the T allele for episodic memory functioning in three separate samples (Papassotiropoulos et al., 2006), multiple studies have supported the notion that the *KIBRA* T allele provides a small but significant benefit to episodic memory and working memory performance (reviewed in a meta-analysis by Milnik et al., 2012). The *KIBRA* gene is located on chromosome 5 and is responsible for producing proteins expressed in the kidneys and brain, from which the name KIBRA is derived (Schneider et al., 2010). A common single nucleotide polymorphism (SNP) occurs within the ninth intron of the gene (reference sequence number 17070145; rs17070145), resulting in a single base (T → C) substitution and producing three possible allele combinations: CC, CT, or TT (Papassotiropoulos et al., 2006; Schneider et al., 2010). The interaction between KIBRA and protein kinase C zeta, which is expressed in the hippocampus and is implicated in long term potentiation (Johannsen et al., 2008; Sacktor, 2008; Lee et al., 2013; Volk et al., 2013; Vogt-Eisele et al., 2014), may be the mechanism by which KIBRA affects memory functioning (Büther et al., 2004; Schneider et al., 2010). Bilateral inhibition of protein kinase C zeta in the dorsal hippocampus of adult rats resulted in poorer (24 h) delayed memory on the radial arm maze and water maze tasks compared to controls; working memory was not impacted by protein kinase C zeta inhibition (Serrano et al., 2008). A SNP specific (rs17070145) comparison detected differential gene expression in T carriers and CC homozygotes: T carriers tended to overexpress genes involved in the MAPK signaling pathway, a pathway involved in learning and memory (Piras et al., 2017).

Recent studies have focused on genetic variants as a way to account, at least partially, for age-related variations in cognitive abilities (examples include Harris and Deary, 2011; Bender and Raz, 2012; Meunier et al., 2014). Regarding *KIBRA* specifically, Milnik et al.’s (2012) meta-analysis found a consistent advantage for T carriers compared to CC homozygotes on episodic memory performance over a wide range of ages (18–100 years of age). Papassotiropoulos et al. (2006) observed effects of *KIBRA* on both verbal and visual episodic memory. However, *KIBRA*-related differences were only detected for a visual memory task among those with psychosis while *KIBRA*-related differences were only predictive of verbal memory among healthy controls (Vassos et al., 2010). Similarly, a TT homozygote advantage over C carriers on verbal memory recall was evident in healthy siblings of those with early-onset schizophrenia, but there was no *KIBRA*-based difference in performance for those with early-onset schizophrenia (Vyas et al., 2014). It is unclear if *KIBRA* has modality-specific effects in memory among older adults. A recent review of the literature suggests that the impact of the T allele increases across the lifespan (Papenberg et al., 2015). This latter finding supports the notion of resource modulation (Lindenberger et al., 2008), which suggests that the influence of genes on cognitive functioning increases with age from young adulthood to middle aged to older adults. For example, some studies have reported that the effect of the *KIBRA* T allele is small in young adults and increases in older adults (Kauppi et al., 2011; Muse et al., 2014).

In contrast, other studies have found little to no effect of *KIBRA* on episodic memory among older adults (Need et al., 2008; Burgess et al., 2011; Laukka et al., 2013; see Schneider et al., 2010 for a review). In one study, for example, Franks et al. (2014) found no differences between T carriers (TTs and TCs) and non-carriers (CCs) in a cohort of 386 healthy aging individuals, ages 50–79, using multiple tests of verbal and visual episodic memory with measures of both immediate and delayed recall. In fact, other studies have even reported a memory *disadvantage* for T carriers compared to CC homozygotes (Nacmias et al., 2008; Wersching et al., 2011).

A potential explanation for the inconsistent results is that the negative impact of other age-related factors may increase variability in memory performance and overwhelm the positive effect of *KIBRA* (Milnik et al., 2012). As an example, Almeida et al. (2008) found that *KIBRA* allele variants have minimal impact once significant cognitive decline is evident among older adults, presumably due to incipient neurodegenerative disease. *KIBRA* studies have increasingly matched allele groups on some of the major factors that may negatively impact memory performance among older adults (Wersching et al., 2011; Boraxbekk et al., 2015; Witte et al., 2016).

One important factor influencing age-related memory is apolipoprotein (*APOE*) ε4 status (Papenberg et al., 2015). The *APOE* ε4 allele not only increases risk for Alzheimer’s disease, but is associated with poorer episodic memory performance in otherwise healthy older adults (Deary et al., 2002; Caselli et al., 2009; Ryan et al., 2011). *APOE* ε4 carriers have smaller hippocampal volumes, on average, compared to ε4 non-carriers (Taylor et al., 2014), and increased age-related changes in white matter diffusion that are associated with poorer memory and executive functioning (Ryan et al., 2011). Given the link between *KIBRA* and hippocampal functioning (Corneveaux et al., 2010; Schneider et al., 2010; Palombo et al., 2013; Schwab et al., 2014; Witte et al., 2016), recent studies have taken both *APOE* and *KIBRA* into account when studying age-related memory changes (Laukka et al., 2013; Palombo et al., 2013; Wang H.F. et al., 2013). Even in these studies, the results are mixed. Laukka et al. (2013) found that including *APOE* status and other genetic variants as covariates removed the effect of *KIBRA*, while others (Palombo et al., 2013; Wang H.F. et al., 2013) found that *KIBRA* still influenced memory performance when groups were matched on *APOE* and other gene variants.

Hypertension is another variable that has negative consequences for age-related cognitive functioning (Raz et al., 2009) and is associated with decreased hippocampal volume (for review, see Beauchet et al., 2013). Few studies have compared *KIBRA* status across groups of older adults matched on hypertensive status. However, one study suggests that *KIBRA* may interact with hypertension status in a complex way (Wersching et al., 2011), such that T carriers who were hypertensive had the poorest performance compared to normotensive T carriers, while both normotensive and hypertensive CC homozygotes performed similar to the normotensive T carriers.

In contrast to risk factors such as *APOE* ε4 status and hypertension, protective factors such as education have also been suggested to impact cognitive functioning in aging. Notably, higher levels of education are associated with better maintenance of cognitive functions even in the presence of early pathology (Stern, 2012). Furthermore, higher levels of education are associated with reduced risk of Alzheimer’s disease (Valenzuela and Sachdev, 2006). Given the potential impact of education on cognition in aging, it is critical to carefully account for this factor in addition to the above-mentioned risk factors.

As noted, *KIBRA*, *APOE*, and hypertension have all been implicated in episodic memory functioning and hippocampal volumes. The hippocampus is especially implicated in episodic memory (Ryan et al., 2001, 2008; Nadel et al., 2007). With regard to *KIBRA*, the evidence is mixed regarding differential patterns of hippocampal function and/or structure between T carriers and CC homozygotes. Using functional magnetic resonance imaging (fMRI), Papassotiropoulos et al. (2006) found increased activation within CC homozygotes greater than T carriers on an episodic retrieval task, suggesting hippocampal compensation for poorer memory performance. Kauppi et al. (2011), however, found an opposite effect on a similar memory retrieval task with greater activation for T carriers relative to CC homozygotes, even when the groups were matched on overall memory performance. Similar inconsistencies apply to structural magnetic resonance imaging (MRI). Papassotiropoulos et al. (2006) failed to detect differences in hippocampal volumes between *KIBRA* groups. Other studies have found larger hippocampal volumes for T carriers compared to CC homozygotes (Palombo et al., 2013; Witte et al., 2016). A recent longitudinal study of older adults failed to detect differential hippocampal volume decline over a 2-year period in T carriers and CC homozygotes (Boraxbekk et al., 2015). Few studies have examined regional brain volumes other than the hippocampus (for example, Wang D. et al., 2013 examined frontal and posterior cingulate regions associated with the default mode network), but to the best of our knowledge, no studies have examined this issue using voxel-based whole brain methods.

The goal of the present study was to characterize age-related differences in cognition and brain volume between *KIBRA* T carriers and CC homozygotes in groups of older adults that were carefully matched on both *APOE* ε4 and hypertension status as well as other demographic variables, so that any differences observed would be more clearly attributable to *KIBRA*. We were also interested in investigating the notion of resource modulation among older adults. Instead of comparing a wide age range of older adults to young adults, we compared across three groups of healthy older adults—middle aged (52–64 years), young old (65–72 years), and older old (73–92 years; Smith et al., 2011). Groups were assessed with measures of episodic memory, executive functioning, and processing speed that have been shown previously to be influenced by *KIBRA*. Following the resource modulation hypothesis, we predicted that the impact of *KIBRA* would be most apparent in the older old group, relative to the middle aged and young old groups. Age-related differences in brain volume were also compared between T carriers and CC homozygotes using voxel-based morphometry. We predicted that the differences in gray and white matter volumes between T carriers and CC homozygotes would increase across the age groups, mirroring the pattern of increased cognitive dysfunction. Last, we hypothesized that the hippocampus would be a region especially impacted by *KIBRA* such that T carriers would have greater hippocampal volumes than CC homozygotes and the difference in volumes would be greatest in the older old group. Multiple imaging studies of *KIBRA* have focused on the hippocampus (Papassotiropoulos et al., 2006; Palombo et al., 2013; Boraxbekk et al., 2015; Witte et al., 2016) given *KIBRA*’s links to episodic memory and hippocampal functioning (Schneider et al., 2010; Milnik et al., 2012).

## Materials and Methods

### Participants

Adults (*N* = 145) between the ages of 52 and 92 years (*M* = 70.61, *SD* = 9.07) who previously participated in aging studies conducted in our laboratories were recruited for the study (Walther et al., 2010, 2011; Ryan et al., 2011; Ryan and Walther, 2014). All participants were living independently in the community. Exclusionary criteria included history of significant head injury, neurological disorder, current or past drug or alcohol abuse, psychiatric disorder, learning disability, the use of antidepressive, antipsychotic, or sleep medications, and contraindications to MRI. Additionally, participants were excluded if they scored below 24 on the Mini-Mental State Examination. Thus, the cohort was relatively healthy with no obvious signs of cognitive dysfunction.

Participants were grouped based on *KIBRA* status as T carriers (TTs and TCs) or CC homozygotes. Demographic information is listed in **Table 1**. *KIBRA* groups were matched on age (*t* < 1) and education [*t*(143) = -1.87, ns], and balanced regarding *APOE* ε4 carrier status [χ^2^(1) = 0.20, ns], gender [χ^2^(1) = 0.20, ns], and hypertensive status [χ^2^(2) = 0.39, ns]. The T carrier and CC groups were then further subdivided into three age groups: middle aged (52–64 years), young old (65–72 years), and older old (73–92 years). Average ages for the three groups were 59.32, 68.09, and 79.36 years, respectively (see **Table 2**). The three groups were well matched on education (*F* < 1). Chi-square analyses indicated that the groups were also similar on *APOE* ε4, *KIBRA*, and hypertensive status [χ^2^s < 3.40, ns] but differed significantly on gender [χ^2^(2) = 8.46, *p* < 0.05]. More specifically, the middle aged group had a lower proportion of males than the young old group [χ^2^(1) = 5.14, *p* < 0.05] and the older old group [χ^2^(1) = 8.44, *p* < 0.05]. Written informed consent was obtained from all participants according to the guidelines approved by the Human Subjects Committee at the University of Arizona and in accordance with the Declaration of Helsinki.

**Table 1:** Participant demographics for *KIBRA* T carriers (TT/TC) compared to CC homozygotes (CC).

	*KIBRA* rs17070145	
	
	TT/TC (*n* = 75)	CC (*n* = 70)
Gender, *n* (M/F)	20/55	21/49
Age (years) *M* (±*SEM*)	70.6 (1.11)	70.7 (1.03)
Education (years) *M* (±*SEM*)	15.3 (0.31)	16.2 (0.33)
Hypertension (%)	24.0	28.6
*APOE* ε4 (%)	22.8	24.6


**APOE* ε4 represents the percent of ε4 carriers (ε4 heterozygotes and homozygotes) in each group. M, male; F, female. All *p*s > 0.05.*

**Table 2:** Demographic information for middle aged, young old, and older old age groups.

	Age group		
	
	Middle aged	Young old	Older old
	(*n* = 38)	(*n* = 45)	(*n* = 62)
Gender, *n* (M/F)^∗^	4/34	14/31	23/39
Age (years), *M* (±*SEM*)^∗∗^	59.3 (0.60)	68.1 (0.31)	79.4 (0.58)
Education (years), *M* (±*SEM*)	15.8 (0.41)	16.1 (0.39)	15.5 (0.38)
Hypertension (%)	21.1	20.0	33.9
*KIBRA* T carriers (%)	67.7	42.2	56.5
*APOE* ε4 (%)	39.5	26.7	22.6


**APOE* ε4 represents the percent of ε4 carriers (ε4 heterozygotes and homozygotes) in each group. M, male; F, female. ^∗^Middle aged adults had significantly fewer males compared to females than did young old and older old adults [χ^2^(2) = 8.46, *p* < 0.05]; ^∗∗^*p* < 0.001.*

### *KIBRA* and *APOE* Genotyping

Saliva samples were collected from each participant using the Oragene DNA Collection Kit (DNA Genotek, Ottawa, ON, Canada) and sent to the Translational Genomics Research Institute in Phoenix, Arizona for genotyping of *KIBRA* rs17070145, *APOE* rs429358, and *APOE* rs7412. Genotyping was carried out using TaqMan allelic discrimination (Applied Biosystems, Foster City, CA, United States) and ABI Prism 7000 sequence detection (Applied Biosystems, Foster City, CA, United States) as described in Corneveaux et al. (2010). *KIBRA* rs17070145 (GenBank accession number NC_000005 GPC_000001297, version number NC_000005.10) genotypes were generated using the KASP chemistry (LGC, Middlesex, United Kingdom). In brief, KASP reactions are comprised of sample DNA, KASP Master Mix, and KASP Assay Mix which contains variant specific probe combinations. The DNA samples are then subjected to polymerase chain reaction to generate fluorescent signals that indicate genotypes for the variants of interest. Amplifications errors resulted in undetermined genotypes for four individuals who were not included in the present study. Genotype distributions for our sample were 0.14, 0.37, and 0.48 for TT homozygotes, TC heterozygotes, and CC homozygotes, respectively. Our sample did not violate Hardy–Weinberg equilibrium [χ^2^(1) = 3.03, *p* = 0.08].

### Neuropsychological Testing

Tests of memory, executive functions, and processing speed were administered as part of a neuropsychological battery described in Glisky et al. (1995) and Glisky and Kong (2008). Memory tests were selected based on their use in previous studies of *KIBRA*. Additionally, tests of executive functions and processing speed were included to determine *KIBRA*’s specificity for memory functioning as previously demonstrated (Papassotiropoulos et al., 2006; Schaper et al., 2008).

The most commonly used memory tests in the *KIBRA* literature involve immediate and delayed word-list recall, story recall, and picture recall. Verbal memory tests included the California Verbal Learning Test (CVLT; Delis et al., 1987) short (∼3 min) and long delay (20 min) word-list recall and the Wechsler Memory Scale-III (WMS-III; Wechsler, 1997) Logical Memory I Story A Recall and Logical Memory II Story A Recall. Visual memory was tested with the Rey–Osterrieth Complex Figure Recall Test long delay (30 min; Bennett-Levy, 1984) and the WMS-III Logical Memory II Faces Recognition Test (Wechsler, 1997). Additional measures of processing speed and executive functioning included Trails A and Trails B from the Trail Making Test (Reitan, 1958) and the total number of words generated beginning with the letters F, A, and S from the Controlled Oral Word Association Test.

### Statistical Analyses

We created composite scores of verbal immediate memory, verbal delayed memory, and visual delayed memory. Composite scores have been shown to be more stable than single tests and increase the power to detect group differences (Crane et al., 2012). Scores from each of the four verbal memory tests were transformed into *z*-scores. Following *z*-transformation, a composite score of verbal immediate memory was created by averaging the CVLT short delay and the WMS-III Logical Memory I Story A Recall test measures. The CVLT long delay and WMS-III Logical Memory II Story A Recall were averaged in the same manner to create a verbal delayed memory composite score. Raw scores on the Rey–Osterrieth Complex Figure Recall Test long delay and WMS-III Logical Memory II Faces Recognition Test were transformed into *z*-scores. Following *z*-transformation, these measures were averaged together to create a visual delayed memory composite score. See **Table 3** for average composite scores per *KIBRA* group. In SPSS v. 21 (IBM Corp, released 2012, Armonk, NY, United States), general linear models (GLMs) were conducted in order to test for an interaction between *KIBRA* and age group. In all models, years of education was entered as a covariate.

**Table 3:** Average *z*-scores for neuropsychological composites for *KIBRA* T carriers (TT/TC) compared to CC homozygotes (CC).

	*KIBRA* rs17070145	
	
	TT/TC (*n* = 75)	CC (*n* = 70)
Verbal immediate memory composite, *M* (±*SD*)	0.03 (0.79)	-0.03 (0.88)
Verbal delayed memory composite, *M* (±*SD*)	0.003 (0.85)	-0.003 (0.93)
Visual delayed memory composite, *M* (±*SD*)	-0.04 (0.76)	0.04 (0.83)


*All *p*s > 0.05.*

### Structural MRI Acquisition and Analyses

High-resolution T1-weighted structural images were acquired on a GE 3T Signa VH/I whole body echospeed magnet and an eight-channel phased array coil (HD Signa Excite, General Electric, Milwaukee, WI, United States) using a 3D spoiled gradient-echo (SPGR) pulse sequence (0.7 mm sections, no skip, TR = 5.1 ms, TE = 2 ms, TI = 500 ms, flip angle = 15°, matrix = 256 × 256, FOV = 260 × 260 mm^2^). Voxel-based morphometry (VBM; Ashburner, 2007) using DARTEL was carried out in SPM8 (http://www.fil.ion.ucl.ac.uk/spm/) following the methods of Ashburner and Friston (2000). Briefly, whole brain SPGR volumes were aligned manually along the anterior to posterior commissural plane. Aligned images were then resliced to 1 mm^3^ and segmented into gray matter, white matter, and cerebrospinal fluid (CSF) and non-brain tissue was removed. DARTEL was used to create a study-specific custom template. Each participant’s gray and white matter images were then normalized to the study-specific template, modulated using Jacobian determinants, and smoothed with a 10 mm full-width-half-maximum isotropic Gaussian kernel. To correct for differences in brain size, intracranial volumes (ICV) were measured by summing gray matter, white matter, and CSF for each participant.

Age-related volumetric changes in T carriers were compared to CC homozygotes (i.e., the interaction between *KIBRA* and age), controlling for ICV, using a regression analysis with *KIBRA* as a categorical factor and age and ICV entered as continuous mean-centered variables. Significance was set at *p* < 0.001, uncorrected, with a conservative minimum cluster size of 50 voxels (Walther et al., 2011). For subsequent analyses, mean cluster volumes were extracted from significant regions of interest (ROIs) for each participant using MarsBaR (Brett et al., 2002). Hippocampal ROIs were identified in PickAtlas 2.0 (Maldjian et al., 2003) with templates based on the Automated Anatomical Labeling library (AAL; Tzourio-Mazoyer et al., 2002). MarsBaR (Brett et al., 2002) was used to calculate the gray matter volumes of the left and right hippocampus for each participant. Left and right hippocampal volumes were also averaged together to obtain a bilateral hippocampal volume.

## Results

### *KIBRA* and Cognitive Measures

GLMs were conducted separately on cognitive measures including verbal immediate memory, verbal and visual delayed memory, Trails A and B, and FAS total score. *KIBRA* status (T carriers, CC homozygotes) and age group (middle aged, young old, older old) were entered as categorical variables. In all models, years of education was entered as a continuous covariate. In particular, we were interested in the interaction between *KIBRA* and age group. The main effect of *KIBRA* was not significant for any of the cognitive measures (all *F*s < 1.67, ns). As expected, the main effect of age was significant for all cognitive measures (*F*s < 22.22, *p*s < 0.001) except for FAS total score [*F*(2,138) = 1.68, ns]. Education significantly predicted FAS total and Trails B performance (*F*s < 11.26, *p*s < 0.001) but did not predict performance on Trails A or any of the composite measures of memory (*F*s < 1).

### Genotype × Age Interaction

**FIGURE 1:**
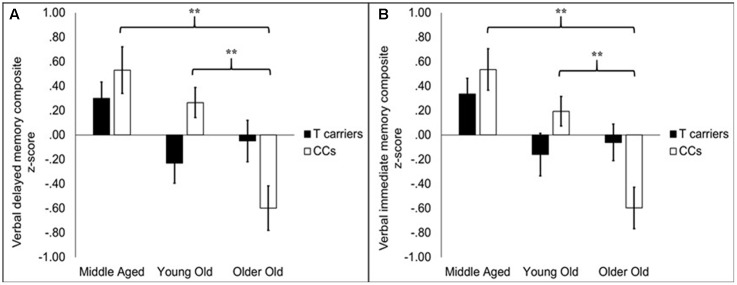
Bar graphs depicting average composite scores on delayed **(A)** and immediate **(B)** memory for *KIBRA* groups broken into age groups. Error bars reflect standard error of the mean. On both measures, performance declined with age group in the CC homozygotes (CCs) but did not significantly decline in the T carriers. In the CC homozygotes, older old participants had significantly lower performance compared to middle aged and young old participants. The latter two groups did not differ. ^∗∗^*p* < 0.001.

While there was no main effect of *KIBRA* on memory, *KIBRA* by age group interactions were evident in both verbal memory immediate and delayed composite scores, as depicted in **Figures 1A,B**, respectively. For verbal immediate memory, the impact of age differed depending upon *KIBRA* status, indicated by an interaction effect between the two factors [*F*(2,138) = 4.58, *p* < 0.05]. One-way follow-up ANOVAs carried out on T carrier and CC homozygote groups separately showed a main effect of age group among CC homozygotes [*F*(2,67) = 13.72, *p* < 0.05] but not T carriers [*F*(2,72) = 2.42, ns]. Among CC homozygotes, middle aged and young old groups performed similarly to one another [*t*(41) = 1.68, ns], and both groups performed significantly better than the older old group (*t*s > 3.77, *p*s < 0.001).

A similar pattern was observed for verbal delayed memory scores. Age groups differed in performance depending on *KIBRA* status [*F*(2,138) = 5.29, *p* < 0.01]. One-way follow-up ANOVAs indicated that age groups differed among CC homozygotes [*F*(2,67) = 12.68, *p* < 0.001] but not T carriers [*F*(2,72) = 2.13, ns]. Verbal delayed memory performance was comparable for the middle aged and young old groups [*t*(41) = 1.23, ns] but was significantly lower among older old adults (*t*s > 3.90, *p*s < 0.001). We further considered whether the associations between our verbal memory composites and the *KIBRA* by age interaction were more attributable to item versus story memory measures within the composite. A mixed-factor ANOVA (*KIBRA* status × age group × test type) showed that both tests (item and story) were influenced similarly by age-related differences in *KIBRA* status, indicated by the lack of interactions between test type and age, *KIBRA*, or their interaction (*F*s < 1).

In contrast to the verbal memory scores, the interaction between *KIBRA* and age group did not approach significance for visual delayed memory or other measures of executive functioning and processing speed (*F*s < 1.76, ns).

### VBM Analyses

**FIGURE 2:**
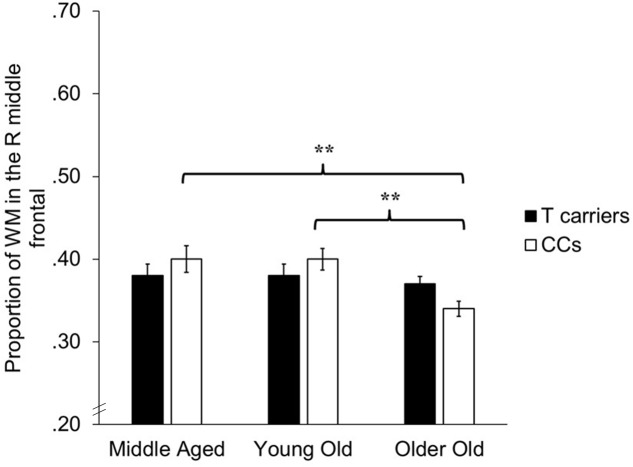
Bar graph depicting average proportion of white matter in the right middle frontal gyrus for T carriers and CC homozygotes across the three age groups. ^∗∗^*p* < 0.001, *k* > 50.

The VBM analyses focused on identifying whether age-related decreases in gray and white matter differed depending upon *KIBRA* status, indicated by an interaction effect between age and *KIBRA* status. The GLMs revealed a region of white matter in the right frontal lobe, adjacent to the right middle frontal gyrus, in which CC homozygotes showed significantly smaller white matter volumes across age groups compared to T carriers [*t*(143) = 3.45, *p* < 0.001, *k* = 81]. **Figure 2** shows the mean volumes in right middle frontal gyrus for T carriers and CC homozygotes by age group. No interactions met significance in gray matter regions, nor did any region of gray or white matter show an age-related advantage for CC homozygotes over T carriers.

### *KIBRA* and Brain Volumes among the Older Old

The *KIBRA* by age interaction in white matter volume occurred primarily because of lower volumes among the older old subgroup of CC homozygotes. Because the whole brain analysis was restricted to interaction effects across the age groups at a relatively conservative statistical threshold, it is possible that we did not see the true extent of *KIBRA*-related volume differences among the older old. We therefore conducted a *post hoc* GLM analysis to assess the impact of *KIBRA* on gray and white matter volumes specifically among the older old group, controlling for age and ICV and employing the same significance threshold (*p* < 0.001, minimum extent 50 contiguous voxels). Note that age was added to this analysis as a covariate along with ICV in order to isolate the effect of *KIBRA*.

**FIGURE 3:**
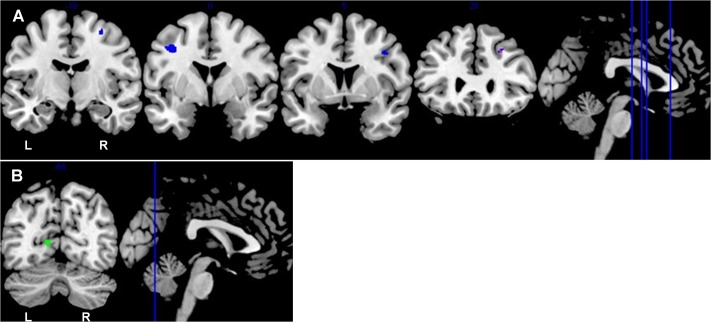
Coronal sections depict areas of *KIBRA*-related differences in white matter **(A)** and gray matter **(B)**, controlling for ICV (*p* < 0.001, *k* > 50). **(A)** Blue represents the subsample analysis depicting regions in which older old T carriers had greater white matter volumes than CC homozygotes. Purple represents an overlapping region in the full sample analysis and the subsample analysis in which older old T carriers had greater white matter volumes than CC homozygotes. **(B)** Green represents the gray matter region in which older old T carriers had greater volumes than CC homozygotes. The locations of the coronal sections are indicated on the accompanying sagittal sections. L, left hemisphere; R, right hemisphere.

Among the older old group, CC homozygotes had smaller brain volumes compared to T carriers in five brain regions (see **Table 4** and **Figure 3**). These included a region of gray matter volume in the left lingual gyrus and four white matter regions in the frontal lobes. Two significant white matter regions were adjacent to the right middle frontal gyrus; other regions were adjacent to the left middle frontal gyrus and right superior frontal gyrus. No regions of gray or white matter were found in which older old CC homozygotes had larger volumes than T carriers.

**Table 4:** Regions in which T carriers showed greater volumes than CC homozygotes, *k* ≥ 50, *p* < 0.001, among the older old group, controlling for age and ICV.

Region	MNI coordinates			*t*-Value (peak intensity)	Cluster size
			
	*x*	*y*	*z*		
**Gray matter region**					
L lingual gyrus	-12	-68	9	3.44	92
**White matter regions**					
R middle frontal	42	5	37	3.80	56
	26	26	40	3.57	111
L middle frontal	-40	1	41	3.72	459
R superior frontal	30	-8	58	3.54	70


*The Montreal Neurological Institute (MNI) coordinates of the location of maximal significance, the *t*-value, and cluster size (in mm^*3*^) of each cluster are provided. L, left hemisphere; R, right hemisphere.*

### Hippocampus

Gray matter hippocampal volumes were extracted and the residuals were calculated after accounting for ICV. Neither *KIBRA* status nor the interaction between *KIBRA* and age predicted measures of left, right, or bilateral hippocampal volumes (*F*s < 1). Age was marginally associated with hippocampal volumes (*F*s > 2.40, *p*s > 0.09).

## Discussion

Our primary objective was to characterize age-related differences in the effect of *KIBRA* on cognition and brain volumes. Our study showed that among a sample of cognitively healthy middle aged, young old, and older old adults, the influence of *KIBRA* on verbal memory performance increased with age, with differences between T carriers and CC homozygotes evident only among the older old group. *KIBRA* also interacted with age to influence gray and white matter volumes in several brain regions, especially the deep white matter of the frontal lobe. Contrary to our hypothesis, hippocampal volumes were not influenced by *KIBRA*. Importantly, these effects were evident in a group of otherwise cognitively healthy adults who were carefully matched on two potentially confounding variables that influence cognitive functioning, namely *APOE* ε4 status and hypertensive status, as well as education. Our findings are broadly consistent with the notion of resource modulation (Lindenberger et al., 2008), where the influence of a genetic variant increases across the adult age range. Below, we compare our study to previous findings in the literature.

### Cognitive Influences of *KIBRA* on Verbal Memory

Collapsed across all older adults in our study, ages 52–92, *KIBRA* had no apparent effect on cognitive measures, consistent with several other null findings reported by Need et al. (2008), Franks et al. (2014), and Boraxbekk et al. (2015). The lack of a primary effect of *KIBRA* should not be that surprising, however. A particular gene may only account for 1–2% of the variance in cognition (Harris and Deary, 2011), making the influence of a single gene difficult to detect, particularly among healthy individuals. Several genes (e.g., *APOE*, *COMT*, and *BDNF*) that also influence cognitive functioning (Shen et al., 2014) may have stronger effects on cognition than *KIBRA* (Witte et al., 2012; Laukka et al., 2013; but see Palombo et al., 2013).

Additionally, cognitive aging is influenced by multiple interactive factors, such as hypertension (Brickman et al., 2008; Bender and Raz, 2012), that may mask or override genetic influences among older adults. Nevertheless, our results suggest that the influence of *KIBRA* may increase after the seventh decade, consistent with Kauppi et al. (2011) who reported stronger *KIBRA* effects on verbal memory among a group of older adults compared to middle aged adults. In contrast, Boraxbekk et al. (2015) found no influence of *KIBRA* on memory measures among young old or older old adults, either cross-sectionally or longitudinally. One aspect of the present study that differs from most others is the fact that we chose to focus on groups of older adults who were carefully matched on critical variables influencing memory such as age, education, *APOE* status, and hypertension status, rather than controlling statistically for the impact of these variables. Stratification increases the power to detect change related to the variable of interest, in this case *KIBRA* (Egbewale et al., 2014), and decreases the likelihood that errors occur in estimating the treatment effect when relying on covariance (Miller and Chapman, 2001).

The present study found an age by *KIBRA* interaction for verbal, but not visual memory performance. Among verbal tests, the effect was observed when memory tasks were combined into a single composite score, as well as individually on follow-up tests for both item and story memory at two delays, immediate and delayed recall. Verbal memory tests are the most consistently reported tests that are influenced by *KIBRA* status (Papassotiropoulos et al., 2006; Schaper et al., 2008; but see Need et al., 2008), although some studies have found distinctions between item memory and story memory for verbal materials (Papassotiropoulos et al., 2006; Bates et al., 2009). In contrast, *KIBRA* status was not predictive of performance on visual memory tasks. A benefit of the *KIBRA* T allele for visual memory performance has been observed inconsistently (Papassotiropoulos et al., 2006; Schuck et al., 2013; but see Nacmias et al., 2008; Franks et al., 2014 for null findings).

Similarly, the lack of an effect of *KIBRA* on measures of executive functioning and processing speed in the present study is consistent with previous reports of small non-robust effects on tasks such as phonemic fluency (Schaper et al., 2008; Laukka et al., 2013). The lack of an association between *KIBRA* and executive measures is somewhat surprising given that white matter integrity and white matter volumes have been linked to processing speed and executive functions (Ryan et al., 2011; Lövdén et al., 2013; Ryan and Walther, 2014), and the present study detected differences in white matter volumes. However, it may depend on the type of executive function being tested. A meta-analysis by Milnik et al. (2012) suggests that the *KIBRA* T allele is consistently associated with working memory, which was not assessed in the present study. Further, *KIBRA* status may be impacting executive functions and processing speed in the context of both age and vascular risk (Wersching et al., 2011). Although Wersching et al. (2011) examined word fluency and complex processing speed using tasks similar to those in the present study (i.e., letter fluency, Trails Making Task), they created composites of word fluency and executive functions/psychomotor speed which also incorporated tasks not administered in the present study (e.g., inhibitory control tasks). Perhaps *KIBRA* has more global rather than specific effects on executive functions.

### *KIBRA* Influences on Brain Volumes

Similar to our cognitive results, brain volumes were impacted by *KIBRA* status depending on age group. The most robust finding was within the white matter of the right middle frontal gyrus: CC homozygotes showed age-related declines in white matter volume in this region compared to T carriers who maintained volumes with increasing age. Wang D. et al. (2013) also observed *KIBRA*-related differences in volumes in specific frontal regions, including medial prefrontal cortex and bilateral anterior cingulate cortex. However, unlike Wang D. et al. (2013), volume changes were observed only in frontal white matter but not cortical gray matter. Declines in brain structure and metabolism in frontal regions have been linked to both healthy cognitive aging and mild cognitive impairment (Loessner et al., 1995; Davatzikos et al., 2008; Bennett et al., 2010; Giorgio et al., 2010). Our results suggest that T carriers may be protected against normal age-related declines in white matter volume in frontal regions.

Further analyses confined to the older old group demonstrated that *KIBRA* differences may be more widespread in frontal white matter among this age group. White matter volumes in bilateral middle frontal gyrus and the right superior frontal gyrus were greater for T carriers compared to CC homozygotes. No gray or white matter region showed greater volumes for CC homozygotes compared to T carriers. Frontal regions of the brain tend to show greater age-related declines than temporal and occipital regions (Resnick et al., 2003), yet *KIBRA* accounted for a significant amount of the variance in frontal volumes after controlling for age.

In contrast to white matter, only one region of gray matter was influenced by *KIBRA* status. Among the older old group, T carriers showed greater gray matter volume in the lingual gyrus compared to CC homozygotes, which may have contributed, at least in part, to their poorer memory performance. The lingual gyrus facilitates autobiographical recollection, possibly through recall of sensory perceptual details (Gilboa et al., 2004; Renoult et al., 2012) and has been implicated in encoding of novel scenes (Hayes et al., 2007). Age-related changes in lingual gyrus volume are also associated with poorer encoding of novel associations (for example, face–name associations; Kalpouzos et al., 2012). Interestingly, white matter integrity in the lingual gyrus may differentiate older adults with mild cognitive impairment from cognitively healthy older individuals (Cooley et al., 2015).

While volumetric changes in lingual gyrus have not been reported previously, several other studies have highlighted *KIBRA*-related changes to posterior midline cortices. Corneveaux et al. (2010) reported reduced glucose metabolism among CC homozygotes compared to T carriers in regions adjacent to the lingual gyrus, including the posterior cingulate and precuneus, as measured by FDG PET, regions previously associated with preclinical risk for Alzheimer’s disease (Davatzikos et al., 2008). Using fMRI, Wang D. et al. (2013) observed increased synchronization of fMRI signal between posterior cingulate and medial prefrontal cortex in young adults with one or more C alleles compared to T-homozygotes. The posterior cingulate has been described as a major “hub” in the default mode network, reflecting a synchronized pattern of resting state activation across the brain (Barkhof et al., 2014). While Wang D. et al. (2013) failed to detect *KIBRA* allele group differences in gray matter volumes in the same posterior cingulate region, they found greater gray matter volumes in medial prefrontal cortex for T carriers compared to CC homozygotes, a region that is directly connected to posterior cingulate within the default mode network. To our knowledge, the influence of *KIBRA* on brain connectivity has not been examined in older adults.

Given the connection between *KIBRA* and hippocampally mediated episodic memory (Schneider et al., 2010; Milnik et al., 2012) and the present finding of impaired verbal memory among older old CC homozygotes, it was somewhat surprising that no differences in left, right, or combined hippocampal volumes between T carriers and CC homozygotes were observed in the present study. This null finding is contrary to recent volumetric studies showing decreased hippocampal volumes among CC homozygotes (Palombo et al., 2013; Witte et al., 2016). However, others have not detected such differences, notably, Papassotiropoulos et al. (2006). A recent longitudinal comparison of hippocampal volumes in older adult *KIBRA* T carriers and CC homozygotes (Boraxbekk et al., 2015) found no differences in hippocampal volumes between T carriers and CC homozygotes at baseline or at 2-year follow-up.

These outcome differences may have arisen because the effect of *KIBRA* is not uniform across hippocampal subregions. Both studies with positive findings (Palombo et al., 2013; Witte et al., 2016) utilized hippocampal subregion analyses. For example, among older adults (ages 50–80), Witte et al. (2016) found robust decreases in CA2/3 and the dentate gyrus among CC homozygotes compared to T carriers, but not for CA1, the subiculum, or total hippocampal volume. Palombo et al. (2013) reported similar results (i.e., greater volumes in T carriers compared to CC homozygotes) among young adults in CA2/3 and dentate gyrus as well as CA1, but not the subiculum. In contrast, studies with null findings (the present study; Papassotiropoulos et al., 2006; Boraxbekk et al., 2015) all confined their examination to total hippocampal volume.

However, it should be noted that differences in sample age-ranges across studies may also contribute to outcome differences. Larger samples among young and older adults combining cross-sectional and longitudinal approaches are needed to better address this issue, and to determine the degree to which *KIBRA*-related differences in hippocampal subregions occur across the adult lifespan. We also note that brain volume may be less sensitive to subtle changes in structure compared to other structural brain measures such as cortical thickness (Hutton et al., 2009; Tucholka et al., 2012) or may be less sensitive to non-linear structural brain changes (Giorgio et al., 2010).

### Limitations

Given our relatively small sample size, we were unable to investigate the impact of other genetic markers associated with cognition (Shen et al., 2014). Second, the variability in age ranges examined across studies makes it difficult to compare results. Finally, the cross-sectional design in the present study limits our interpretations. We cannot determine whether the age-specific effects of *KIBRA* detected in the present study reflect changes that occur with aging as the resource modulation hypothesis predicts or if the effects are merely a result of cohort differences. Further, our study cannot determine if poorer performance on verbal memory tasks and smaller brain volumes in the CCs results in increased risk for later cognitive impairment compared to T carriers.

## Conclusion

In conclusion, our findings support the resource modulation hypothesis (Lindenberger et al., 2008). Even in a cohort of relatively healthy and high functioning older adults who were carefully matched on many factors associated with cognitive function and/or brain volume, including age (Hedman et al., 2012), education (Tucker and Stern, 2011; Stern, 2012), gender (Resnick et al., 2000; Jack et al., 2015), hypertensive status (Beauchet et al., 2013), and *APOE* ε4 status (Taylor et al., 2014), poorer verbal memory and smaller brain volumes were associated with the *KIBRA* CC allele subgroup, particularly among older adults in their Seventh decade and beyond. Our findings warrant longitudinal investigation into other measures of brain structure which may be mediating the impact of *KIBRA* on cognition.

## Author Contributions

AS contributed to study design, performed neuroimaging analysis, performed statistical analysis, interpreted the data, and drafted the manuscript; KK contributed to study design, performed neuroimaging analysis, and drafted the manuscript; KW collected the neuroimaging and cognitive data, contributed to neuroimaging analysis procedures, and critically reviewed the manuscript; EG contributed to interpretation of statistical analysis and critically reviewed the manuscript; MH contributed to study design, performed genotyping, and critically reviewed the manuscript; RR performed genotyping and reviewed the manuscript; LR contributed to study design, interpretation of neuroimaging and cognitive data, and drafted and reviewed the manuscript.

## Conflict of Interest Statement

The authors declare that the research was conducted in the absence of any commercial or financial relationships that could be construed as a potential conflict of interest.
